# The complex adolescent world that complicates preventive care in GP consultations: a qualitative study

**DOI:** 10.3399/BJGPO.2024.0290

**Published:** 2025-09-10

**Authors:** Elodie Million, Manon Herbreteau, Gérard Bourrel, Bruno Falissard, François Carbonnel, Béatrice Lognos, Agnès Oude Engberink

**Affiliations:** 1 Department of General Practice, University of Montpellier, Montpellier, France; 2 University Pauline Lautaud Multiprofessional Health Centre, Saint-Georges-d’Orques, France; 3 Desbrest Institute of Epidemiology and Public Health, University of Montpellier, Montpellier, France; 4 Centre de Recherche en Epidémiologie et Santé des Populations, Institut National de la Santé et de la Recherche Médicale U1018, Hôpital Paul Brousse, Université Paris-Saclay, Villejuif Cedex, France; 5 Avicenne Multiprofessional Health Centre, Cabestany, France

**Keywords:** adolescent, prevention, primary health care, general practitioners, mental health, sexual health

## Abstract

**Background:**

Preventive care is important to the international primary care system. In adolescents, several prevention areas need to be addressed; for example, sexual health, mental health, substance use and addiction, physical activity, screen use, and social relationships. However, consultations with adolescents are complex, which puts health professionals in a difficult position. While there are professional recommendations in many countries, they focus on a single theme.

**Aim:**

To understand the preventive approaches taken by GPs in consultation with adolescents.

**Design & setting:**

This was a qualitative study using semi-structured interviews with French GPs.

**Method:**

Participants were recruited following direct requests and sampled using the snowball sampling method. Analysis was conducted using grounded theory for the identification of conceptualising categories.

**Results:**

Twelve interviews led to the emergence of the following four conceptualising categories: 1) the characteristics of adolescents make the preventive approach complex and generate a fear of failure for GPs; 2) the world of the adolescent is foreign to the GP, which is detrimental to developing an empathic relationship between them, negatively impacting prevention; 3) the GP, as an individual, approaches adolescent prevention in the context of their own experiences (parenthood, their own adolescence, and professional practices) and interpersonal skills; and 4) GPs propose an optimised prevention approach for adolescents.

**Conclusion:**

GPs are opportunistic in terms of providing preventive care for adolescent patients. Any occasion for consultation should be a chance for a preventive action, whatever the initial motive. GPs require tools and consultations dedicated to prevention to optimise their approaches. A further qualitative study looking at the experiences of adolescents seen by their GPs would be an interesting way of completing our understanding of the prevention carried out during these consultations.

## How this fits in

Primary healthcare professionals should address all areas of preventive care when consulting with adolescents, but it can be complex and put health professionals in a difficult position. The GPs in our study confirmed these prevention difficulties. To improve this primary and often opportunistic prevention during consultations with an adolescent, GPs recommended the use of a pre-consultation questionnaire before a dedicated prevention consultation, a regularly updated dictionary of the adolescent world, and an easy-to-use digital tool in the consultation.

## Introduction

Adolescence is a period of transition from childhood to adulthood, and is more than just a biological stage defined by the onset of puberty.^1^ The desire for autonomy and experimentation, need to identify with peers, and risk-taking are all psychosocial changes observed during adolescence.^1–3^ It is also a period of vulnerability that requires the identification of risky behaviours to prevent them.^4,5^ Sexual health, mental health, substance abuse and addiction, diet, physical activity, orthopaedic disorders, vaccinations, screen use, and social relations are some of the prevention areas that need to be targeted.^6–9^ A 2010 French survey revealed that 80% of the 6004 adolescents questioned had consulted a GP over the past 12 months, with an average of 2.3 consultations per year.^6^ During consultations, GPs talked about prevention, even if the initial reason for consultation was unrelated; nearly one in two consultations was related to prevention for those aged 13–24 years.^7,10^ Prevention also plays an important role in primary care in other countries such as in Europe and North America.^11–13^ Consultations with adolescents are complex,^13–17^ which makes it difficult for health professionals to take preventive action. It is important that communication is adapted to the adolescent’s level of maturity. Specific professional relational skills are needed, and there are multiple areas of prevention to be taken into account. There can also be fluctuations in the adolescent’s identity, which is a major issue in the care of adolescents because of the consequences for their adult lives.^14,17,18^ Further, the presence of an accompanying third party and the short consultation time makes it difficult for GPs to cover all areas of prevention and specificities of communication with adolescents.^13–16,18^ A 2021 French study showed that certain areas of prevention, particularly addiction, mental health, and nutrition, were neglected during general medical consultations.^7^ Other areas of prevention are sports, vaccination, and contraception.^7^ Early identification of risky adolescent behaviours is essential for reducing morbidity and mortality.^15,19^ There are recommendations and a variety of tools on prevention for adolescents in consultations.^8,9,17,20^ However, these recommendations and the literature essentially focus on a single theme, usually mental health^19,21–24^ or sexual health.^25–28^ Healthcare professionals should ideally address all areas of preventive care when consulting with adolescents.

We, therefore, plan to create a digital tool to help with preventive care during consultations with adolescents. This tool will be available to doctors and healthcare professionals who see adolescents in consultation. It will make it easier to identify and optimise prevention in all areas of adolescent health. Before creating the tool, however, it was necessary to examine the practices of GPs, considering they see adolescents the most in France. The digital tool will, thus, be more relevant, having been enriched by the practices and needs of professionals in the field.

As such, this is a preliminary study on the creation of a digital tool for prevention in consultation with adolescents. We conducted a qualitative study using semi-structured interviews to understand the preventive approaches adopted by GPs during consultations with adolescents. Our aim is to suggest methods to improve preventive care.

## Method

### Study design

We conducted a qualitative study of French GPs. Semi-structured interviews were conducted in French by one researcher (MH) who received specific training in qualitative interviews. Interviews were translated into English via Elsevier Language Editing. The researcher used an interview guide focused on lived experiences, developed by three experts in qualitative research (GB, AOE, and EM) and pre-tested on a sample of two GPs. The guide is based on literature data, using open-ended questions to gain an in-depth understanding of the GPs’ lived experiences; these were modified during the two initial interviews, and no other subsequent changes were made (Table 1). This approach contributes to the internal validity of this study.

**Table 1.** Interview guide

**Primary question**	**Probing questions**
When I say, ’adolescent prevention’, what comes to mind?	
What is your practice’s approach to adolescent prevention?	How do you approach the adolescent patient? What tools do you use? How do you behave? What is your position in relation to the adolescent and their carer?
Can you tell me about a consultation experience in which you discussed prevention with adolescents?	How did you react in this situation? What emotions did you feel?
In your opinion, what could influence the implementation of adolescent prevention in general practice?	In your opinion, what are the obstacles and levers to prevention for adolescents?
In your opinion, what would be the ideal way to carry out adolescent prevention in general practice?	How can we improve prevention among adolescents in practice? By what means? What would be your expectations of a prevention tool for adolescents aimed at doctors?
In everything you’ve just told me, what aspect do you think is the most important?	

### Participants

French GPs were recruited following direct requests from the two principal investigators (MH and EM) and sampled using the snowball sampling method. This method allowed us to complete the purposive sample, which was otherwise too homogenous. A purposive sample (that is, a targeted sampling of GPs who were competent to answer the research questions) was sought with maximum variation in personal experience (age, sex, parenthood, and children) and professional experience (practice setting and duration, estimated percentage of adolescents in the patient base, university internship master’s degree, working in a multidisciplinary team, and specific training in adolescent health). This variation contributed to the external validity of this study. No payment or financial compensation was provided.

### Data collection

Individual in-depth interviews took place at the location of the interviewees’ choice; all doctors chose the location of their own practice. The interview began with a written information sheet and questionnaire to collect the participants’ characteristics, followed by the investigator obtaining the interviewees’ oral consent to participate in the study and their agreement to be recorded using a smartphone (iPhone X). The recording was destroyed immediately after verbatim transcription. The participants were informed of the study objectives and that they could stop the interview at any time without giving a reason.

### Data analysis

The recorded patient interviews were transcribed verbatim. The text was then analysed using floating reading and annotation. Continuous comparisons enable the identification of conceptual categories, based on a grounded theory model.^29^ Table 2 shows the steps that were taken for the analysis. We aimed to explore and understand the experiences of the GPs rather than perform a theoretical analysis.^29^ No analytical software was used in this study. The analysis was carried out when the interviews were completed, until data saturation was reached. Two further interviews were required to confirm this saturation (the process followed the Consolidated criteria for reporting qualitative research; COREQ). Three researchers (EM, MH, and AOE) triangulated the data employing inductive content analysis, using a process of constant comparison between the text and categories. This strict methodology ensured the internal validity of the study.

**Table 2.** Analysis steps

**Accurate transcription of the recordings** Transcription of interviews Progressive analysis of interviews
**Floating reading** Identification of the most relevant elements of the verbatim reading and annotation
**Open coding** Coding as close as possible to the text to preserve meaning Division text into meaning chunks through focused reading Name first themes
**Axial coding** Back and forth between the data that is already analysed and the data recorded in the new verbatim text being analysed Assembling meaningful and similar elements or elements relating to the same lexical field using an inductive approach between different verbatims
**Categorisation** Horizontal coding: grouping themes for a first categorisation Continuous comparisons between conceptualised categories under construction and the emerging conceptualised categories of the following verbatims Increase in the level of generalisation categories until theoretical saturation
**Meaning of the phenomenon** Arranging conceptualised categories according to their logical inter-relationships to construct the meaning of a phenomenon

## Results

### Participants

All contacted GPs agreed to participate in the study. The interviews were conducted between October 2022 and November 2023. Twelve interviews were carried out with nine females and three males; adolescents accounted for 5%–20% of their patients (Table 3). The interview duration ranged from 20–49 minutes.

**Table 3.** Characteristics of the participants

Identifier	Sex	Age, years	Number of children (ages in years)	Practice environment	Number of years as a GP	Estimated per cent of adolescents in the patient base	Specific training in adolescent health
E1	F	46–55	3 (16, 19, 19)	Rural	10–15	5–10	Yes
E2	F	36–45	3 (8, 13, 15)	Semi-rural	10–15	5–10	Yes
E3	F	56–65	2 (26, 27)	Semi-rural	>20	5–10	No
E4	F	<35	1 (1)	Semi-rural	<5	11–20	Yes
E5	F	<35	0	Semi-rural	<5	5–10	No
E6	M	36–45	2 (3, 5)	Semi-rural	10–15	5–10	No
E7	F	36–45	2 (8, 13)	Rural	10–15	11–20	No
E8	M	<35	1 (1)	Urban	5–10	<5	Yes
E9	F	46–55	2 (10, 23)	Semi-rural	15–20	5–10	No
E10	M	46–55	4 (5, 8, 21, 24)	Urban	15–20	<5	Yes
E11	F	36–45	2 (9, 14)	Semi-rural	10–15	5–10	No
E12	F	56–65	2 (25, 28)	Semi-rural	>20	<5	Yes

F = female. M = male.

### Conceptualising categories

Four conceptualising categories emerged from the verbatim transcriptions. The four categories are discussed below and all of the subcategories are given in Table 4.

**Table 4.** Summary of conceptual categories

Category	Subcategory
The characteristics of adolescents make the preventive approach complex and generate a fear of failure for GPs	Prevention among adolescents is mostly primary prevention, with actions to promote ’good health’ before identifying adolescents at risk
	There are specific characteristics of this vulnerable adolescent group and risk-taking period
	Prevention in adolescents by GPs is opportunistic
	The accompanying parent has an important role to play
The world of the adolescent is foreign to the GP, which is detrimental to developing an empathic relationship between them, negatively impacting prevention	The GP does not perceive adolescence as a transition period distinct from childhood and adulthood
	The teenage world is difficult to understand for the GP
	Communication between GP and adolescent is difficult
The GP, as an individual, approaches adolescent prevention in the context of their own experience (parenthood, their own adolescence, and professional practices) and interpersonal skills	The age and sex of the GP plays a role in certain areas of prevention
	The family’s position towards the attending physician can be both a facilitator and a hindrance to the prevention process with the teenager
	The GPs use their varied personal interpersonal and communication skills to optimise their preventive discourse with adolescents
	The GP’s personal experience influences the prevention and communication they established with the adolescent
GPs propose an optimised prevention approach for adolescents	A consultation with the GP dedicated to prevention is recommended
	A pre-consultation questionnaire is essential to the effectiveness of these prevention consultations
	GPs requested tools that could be used during consultations with adolescents

#### The characteristics of adolescents make the preventive approach complex and generate a fear of failure for GPs

Prevention in adolescents by GPs is a type of opportunistic primary prevention. The multiple exposures to risk, the vulnerability, and the specific communication and consultation characteristics of the adolescent make the preventive approach complex and generate a fear of failure among GPs.

The GPs listed the risks to which adolescents might be exposed as influenced by national prevention campaigns (obesity, vaccines, and screen use). They then described the obstacles in tackling all preventive themes in a consultation, considering the lack of time or mastery of the subject, prejudices, and selection of only those themes deemed the most important (prevalence or potential seriousness):

*‘When you have 15 minutes to discuss all these subjects with a teenager, you inevitably make choices regarding the questions to ask and you skip over certain things.’* (E11)

*‘Drugs are a subject I don’t master, so I don’t really like to talk about it.’* (E5)

GPs validated the importance of preventive health care for adolescents given the specific characteristics of this vulnerable and risk-taking group. However, communication difficulties generated a fear of not recognising an adolescent in danger:

*‘We know that this is a fragile age, with a high risk of suicide and of being manipulated by other people.’* (E3)

*‘Even if we see him just once and ask him how he’s doing, if he feels like saying yes even though he’s crying in bed every night and hiding it from everyone, well, we’re missing out.’* (E5)

Even if adolescents consulted the GPs for short periods for an acute pathological reason, the doctors seized every opportunity to discuss prevention. More targeted reasons for consultation made it easier to address issues such as sexual health, suicide risk, and substance abuse. The best opportunity to discuss preventive care with adolescents was during consultations to determine whether they had any contraindications to practising sports. However, some adolescents did not consult with GPs, making the opportunistic prevention approach impossible:

*‘Today, we take advantage of a reason for consultation to initiate these prevention topics.’* (E6)

*‘If you don’t have any health problems, don’t do any sports, don’t complain about anything … there’s no reason for your parents to take you to the doctor.’* (E12)

While the accompanying parent played an important role in adolescent prevention, the triangular relationship (parent, doctor, and adolescent) made the GP’s approach to prevention more complex. The parent could be an ally, enriching dialogue in consultation and continuing actions at home. Some doctors even described the parent’s predominant role in medical decisions, with the adolescents being consulted and their agreement sought, but not regarded as obligatory. However, parents’ behaviour could also be detrimental to communication with adolescents; time alone with adolescents is necessary, even if the GP faces difficulties in getting the parent out of the consultation:

*‘When you’re an adolescent, you understand a lot of things, and you can already do something about your health, but you’re not the one running the errands or organising your home. That’s why parents also need to get involved.’* (E5)

*‘Sometimes they* [the parents] *are in a hurry or worried, they will speak for their child without ever letting them speak.’* (E11)

#### The world of the adolescent is foreign to the GP, which is detrimental to developing an empathic relationship between them, negatively impacting prevention

GPs do not understand the world of adolescents, which leads to communication difficulties and a feeling of inefficiency during consultations, especially when intimate subjects are discussed and the adolescent is young.

The GPs felt overwhelmed, and tried to keep abreast of the cultural specificities of adolescents to facilitate communication. They described the adolescent preoccupations that were at odds with their adult vision: a strong emphasis on physical appearance; desocialisation of certain adolescents despite being in continuous digital connection with the world; shocking behaviour or discourse; and sometimes irritating the doctor owing to a lack of empathic understanding. GPs sometimes found it difficult to find a place in the care of adolescents who are not doing well because of their lack of understanding of the codes of their social world:

*‘At that age, they have trouble understanding that what they do today can have an impact on their future health. Teenagers are abrupt, they don’t think about tomorrow.’* (E9)

*‘*[Adolescents] *are totally on another planet! We can’t understand each other.’* (E7)

The GPs did not perceive adolescence as a transition period distinct from childhood and adulthood. Consequently, they felt uncomfortable discussing certain prevention topics with younger patients, for fear of offending them. However, the GPs played a role in the adolescent transition, whereby the repeated preventive messages from physicians who had treated the patient since childhood helped them develop into a future adult:

*‘That’s also the exciting thing about general practice: you get to see patients evolve over the long term, you get to see children become adults, life itself!’* (E10)

The GPs who refused to discuss a certain subject, and feared lack of honesty on the part of the adolescents in discussions, were worried about a breakdown in dialogue if the discussed subject displeased the adolescents; or that the adolescents felt afraid of being judged or reported to parents. These communication difficulties could damage the relationship between the GP and adolescents, generating feelings of powerlessness, fear of not spotting adolescents in trouble, and even feelings of exhaustion and annoyance. The GPs saw male adolescents as less communicative and less receptive to discussions than their female counterparts:

*‘*[Adolescents] *don’t answer me, or provide closed answers. Typically, the adolescent looks at his shoes, head down, waiting for it to pass.’* (E7)

*‘I felt a bit helpless, I couldn’t find the right angle to engage in a discussion with this adolescent.’* (E6)

#### The GP, as an individual, approaches adolescent prevention in the context of their own experience (parenthood, their own adolescence, and professional practices) and interpersonal skills

The GP’s personal characteristics — such as the age and sex of the practitioner and whether they are a doctor and the parent of an adolescent — influence the relationship with the adolescent and, subsequently, the prevention implemented. The relational skills used by GPs vary depending on the issues addressed.

The age and sex of the GP played a role in certain areas of preventive care. Prevention messages were easier and better received when adolescents could identify with or relate to their GP (same sex), particularly when sexual health and puberty were discussed. Among doctors, adolescents were more receptive to what a young GP had to say, felt understood, and judged less:

*‘Adolescents … I have the impression that they are more at ease when the doctor is young* [...] *as if we could understand a little better because we went through it not so long ago.’* (E5)

The family’s position towards the attending physician could be both a facilitator and a hindrance to the prevention process with the adolescent. The attending physician was often familiar with the adolescents’ family system and social environment, which facilitates prevention. They could thus approach prevention in a progressive and repeated way, consistently adapting to the adolescent’s age. However, this repetition of prevention messages, with no certainty of effectiveness, diminished attending physicians’ motivation. The confidentiality of the consultation could be questioned by adolescents when they talk to the family GP. Finally, the GP may have some difficulty getting a parent out of the consultation because they had been their practitioner for a long time:

*‘That’s the advantage of the family doctor. If you’ve always been there and you’ve always listened to him, it’s easier for you to be there when things aren’t going well actually.’* (E3)

*‘It’s frustrating* [...] *I feel like I’m always telling them the same thing, trying to get messages across, and the next time nothing has changed.’* (E7)

The GPs used their own interpersonal and communication skills to optimise the preventive discourse with adolescents. Not all GPs had the same skills, and several techniques were used: knowing and targeting the topics that interested the adolescents; asking open but precise and direct questions; and using standardised tools. Other communication approaches include motivational interviewing, the use of humour and adolescent codes, and the use of familiarity to create closeness:

*‘I try to talk to them in a simple, straightforward way. Innuendo and all that doesn’t work with teenagers!’* (E9)

The GP’s personal experience influenced the prevention and communication they established with the adolescents during consultation. Being an adolescent parent gave the GP a better understanding of the issues; however, there was a risk of projecting from their personal life. When discussing certain risky behaviours with adolescents, the GP could feel dishonesty in their discourse because of similar personal experiences:

*‘I can’t see myself telling them "it’s not right" when not so long ago I was doing the same thing … I don’t feel too legitimate.’* (E8)

*‘Children who aren’t doing well* [...] *makes me feel bad for my own children and that can be complicated.’* (E1)

#### GPs propose an optimised prevention approach for adolescents

GPs propose optimising the prevention process for adolescents: one or two compulsory consultations, reimbursed by the health insurance fund, preceded by the completion of a pre-consultation questionnaire; and the development of digital tools to help with risk prevention and communication during consultations.

The GPs recommended a consultation with them dedicated to prevention. They proposed one consultation at age 15 years or two consultations at ages 11–13 years and 16–17 years, to be personalised according to age. These consultations should be compulsory and fully reimbursed. GPs proposed introducing them to adolescents and their families by sending them an email or a letter from the health insurance fund, or through TV or social networking campaigns:

*‘It would be great if there was a prevention consultation for adolescents* […] *That’s a lot for a single consultation for an adolescent, there should be several.’* (E10)

For GPs, a pre-consultation questionnaire is essential to ensure the effectiveness of prevention consultations. The questionnaire must be short (10 questions maximum) and accessible via a smartphone application or a QR code in the waiting room to target the subjects to be discussed and facilitate dialogue.

The GPs also recommended tools that could be used in consultations with adolescents to support the prevention approach and let them speak freely. Business software should help integrate questionnaires to systematise the examination and monitoring of adolescent prevention indicators. Useful resources for adolescent prevention approaches can be grouped on websites or applications. Digital prevention scales can be used by adolescents to assess their risky behaviours without the need to verbalise their problems. An adolescent or adult dictionary can facilitate communication with adolescents. The GPs can offer adolescents information sheets or digital resources related to the prevention topics discussed in the consultation:

*‘A sort of practical guide on how to approach certain subjects with the adolescent, with flowcharts* […] *on subjects* […] *that pose the most problems* … *‘* (E8)

## Discussion

### Summary

The following four conceptualising categories emerged from the 12 interviews with GPs to understand their experience of prevention in consultations with adolescents: 1) the characteristics of adolescents make the preventive approach complex and generate a fear of failure for GPs; 2) the world of adolescents is foreign to the GP, which is detrimental to developing an empathic relationship between them, negatively impacting prevention; 3) the GP, as an individual, approaches adolescent prevention in the context of their own experience (parenthood, their own adolescence, and professional practices) and interpersonal skills; and 4) GPs propose an optimised prevention approach for adolescents.

### Strengths and limitations

The relationship between the GP and the adolescent has already been studied in the literature, but no study, to our knowledge, has specifically looked at the GPs’ experiences of prevention in consultations with adolescents. Qualitative research is especially adapted to exploring these experiences; the qualitative method and grounded theory allowed a deep understanding of the experience of GPs regarding this prevention. Credibility was respected by the methodological choice as triangulation of the analysis by two researchers and the purposive sample. Findings may be transferable to similar settings in countries with the same social or cultural structures.

It would have been interesting for the sample to include other healthcare professionals who treat adolescents. However, in France, GPs see adolescents the most, as adolescents account for only 2% of the procedures carried out by private paediatricians, and only one in five adolescents has seen a school doctor in the past year.^30^

### Comparison with existing literature

According to the interviewed doctors, prevention for adolescents is largely opportunistic. Indeed, they described the reasons for adolescent consultations as often being acute and unrelated to preventive measures. This perception is confirmed by the literature. In France, as in the US and the UK, adolescents consult their GPs mainly for acute pathologies and trust their GPs to treat them.^31,32^ French studies have shown that adolescents are unaware that prevention issues can be discussed during a consultation or believe that the doctor will not have time for it;^14,33,34^ contrastingly, in US and UK studies, adolescents appreciate receiving preventive information during consultations, especially when they feel at ease with their doctor.^31,32^ In France, only 12% of adolescents cited their GP as a reference for prevention regarding mental and sexual health.^6^

The adolescent world was sometimes strange and incomprehensible to the GPs in our study. The GPs felt overwhelmed by the rules governing the adolescent world, especially when they were not in line with the values of the professional. This incomprehension could lead to a lack of empathy and impact on the relationship between the GP and the teenager. To overcome this difficulty, some participants proposed the creation of a dictionary of adolescent/adult language, or being keept up to date with adolescent fashions. However, it is not necessary to know or validate this adolescent world in order to care for an adolescent in a consultation and develop a preventive approach. GPs need to understand how to communicate with the young person while maintaining an empathetic and appropriate communication with the adolescent.^13,14,18^ There are numerous recommendations for good practice in France and worldwide, including verbalisation tools, dedicated consultation time, broadening of the initial reason, and identification tools, particularly for mental health.^13,35–37^ Although many of these tools and recommendations were cited by the surveyed GPs, they had great difficulties in putting them into practice.

In France, as in other countries, several pre-consultation questionnaires have been designed for use in consultations with adolescents.^36,38–40^ Moreover, the HEADSSS is recommended in many countries worldwide as a consultation guide, but has been defined as time-consuming.^38,41^ These pre-consultation questionnaires and HEADSSS guide are mainly used by hospital doctors or specialists (paediatricians and child psychiatrists); few GPs use them in practice because of lack of time, knowledge, or technical feasibility in the office.^38^ One study revealed that adolescents and parents were in favour of its use and that it facilitates the discussion of certain specific problems with GPs.^38^

There are many thematic tools and questionnaires in the extant literature for identifying and preventing adolescent illnesses; however, they are often difficult to use in primary care consultations. The complexity of the consultation, the need for comprehensive risk identification and prevention in adolescents, and the recommendations made by GPs in our study confirm the need for comprehensive and practical digital tools to facilitate the prevention communication with adolescents by primary care physicians. Our study will enable us to refine the content to meet the expectations of healthcare professionals.

In France, GPs are the main healthcare professionals encountered by adolescents. The doctors in our study emphasised the importance of the family doctor’s role in implementing preventive measures for adolescents, which is confirmed in the French literature.^18^ However, the repetition of sometimes ineffective prevention messages exhausted the GP, who described feeling frustrated; this could negatively impact their adolescent-centred approach. One solution may be to move closer to the US prevention model, which relies on a multidisciplinary care network rather than a single doctor.^31,42^ This model would relieve the burden on the GP; however, adolescents could lose the benefit of a single medical contact with whom they have built a relationship of trust over time.

### Implications for research and practice

GPs were involved in the adolescents’ prevention but mentioned that they lacked a practical tool that could be easily used in their consultations. Therefore, our suggestion to create a digital tool that covers all areas of adolescent prevention is relevant. It is also important to understand adolescents’ experiences of prevention through their GP so that doctors can offer an approach tailored to the needs of the adolescents themselves. A digital tool will be needed, but will not be enough, as the experience of the GPs in our study has shown. GPs asked about consultations dedicated to adolescent prevention or pre-consultation questionnaires. Moreover, GPs, like other healthcare professionals involved in the care of adolescents, need to be better prepared to practice motivational interviewing as part of a preventive approach, just as they do with adults. This approach has amply demonstrated its superiority to simple advice or warnings in all preventive approaches, including with young people.^43^
